# A Systematic Review of the Management of Maternal Obesity in Pregnancy: Antenatal Management, Outcomes, and Long-Term Implications on Maternal Health

**DOI:** 10.7759/cureus.87258

**Published:** 2025-07-03

**Authors:** Farrah Mukhtar

**Affiliations:** 1 Obstetrics and Gynaecology, George Eliot Hospital, Nuneaton, GBR

**Keywords:** antenatal care, gestational diabetes, long-term maternal health, maternal obesity, pregnancy outcomes

## Abstract

Maternal obesity is a looming worldwide health challenge with dire consequences regarding pregnancy outcomes and future maternal and offspring health. This systematic review synthesizes evidence from eight high-quality studies published between 2015 and 2024, discussing the management of maternal obesity during pregnancy, antenatal care strategies, associated perinatal outcomes, and long-term maternal health risks. The review identifies a strong correlation between maternal obesity and the increased chances for gestational diabetes mellitus (GDM), hypertensive disorders, preterm birth, cesarean delivery, and neonatal morbidity, including large for gestational age (LGA) infants and congenital anomalies. Moreover, there are long-term maternal impacts of increased chances for type 2 diabetes mellitus and cardiovascular disorders. Also, there is an increased risk of metabolic and neurodevelopmental disorders for the offspring. There are significant differences in the antenatal management strategies in settings with conflicting clinical guidelines on the issues of gestational weight gain, screening protocols, and lifestyle counseling. The early pregnancy holds promises in the improvement of outcomes in multidisciplinary interventions, but implementation is still limited. A predictive risk model based on maternal comorbidities, age, and pattern of fat distribution may provide a foundation for individual treatment and needs to be validated. As outlined in the review, the need for standardized and evidence-based guidelines for coordinated antenatal care is emphasized to reduce the harmful effects of maternal obesity. It is crucial not only for the improvement of maternal and neonatal outcomes but also from the viewpoint of breaking the intergenerational cycle of obesity- related health issues.

## Introduction and background

Maternal obesity has become a major public health issue with significant effects on pregnancy, childbirth, and the long-term health of the mother and offspring [1]. The global rates of obesity in women of reproductive age have been increasing at a steady pace, with multiple factors such as sedentary lifestyle, poor nutrition, and socioeconomic determinants of health playing a key role [World Health Organization (WHO), 2023] [2]. Based on the recent worldwide estimates, about 20-25% of pregnant women are considered to be obese (BMI ≥30 kg/m²), whereas even higher rates are observed in certain high-income countries [3]. This trend has key implications for the field of obstetric care, and there is an urgent need to better understand and manage the risks involved in maternal obesity during pregnancy [4].

Obesity in pregnancy is linked with many maternal and fetal complications [5]. Several studies have shown that maternal obesity is a major risk factor for gestational diabetes mellitus (GDM), hypertensive disorders including preeclampsia, cesarean delivery, and preterm birth [6]. These complications not only cause short-term maternal and neonatal morbidity but also lead to long-term effects that will persist after the perinatal period [7]. For instance, obese women are more likely to suffer from type 2 diabetes in the course of their lives, as well as develop cardiovascular disease and metabolic syndrome [8]. Children of overweight mothers are also more prone to developing obesity, type 2 diabetes, and neurodevelopmental problems, thereby contributing to the intergenerational cycle of health inequality [9].

Furthermore, maternal obesity and adverse outcomes are closely linked with each other; however, there is wide inconsistency in antenatal management strategies in different clinical settings [10]. The existing guidelines vary significantly regarding recommended gestational weight gain, antenatal visit schedule, screening procedures for metabolic diseases, and nutritional and physical activity advice [11]. In many cases, clinical practice fails to capture the intricacy of working with obese pregnancies, at least partially due to knowledge gaps among clinicians, a lack of standard operating procedures, and insufficient provision of multidisciplinary care [12]. Such inconsistency presents obstacles to optimizing maternal and neonatal outcomes and calls for a more integrated, evidence-based antenatal care for obese pregnancies [13]. In addition, maternal obesity poses diagnostic and therapeutic challenges during pregnancy [14]. Obesity may hinder ultrasound and clinical evaluation, leading to delays in the diagnosis of fetal growth abnormalities or structural anomalies [15]. It may also affect the pharmacokinetics of medicines taken during pregnancy and labor, necessitating dose adjustment, which is typically not clearly stated in existing guidelines [16]. These issues make the management of high-risk pregnancies more complex and accentuate the need for early, personalized planning of care.

Given these multiple challenges, the recent literature has highlighted the possible advantages of targeted antenatal interventions. In particular, lifestyle changes encouraged by dietary counseling, physical activity programs, and psychological care when initiated early have demonstrated the possibility of enhancing the outcomes in pregnancy [17]. Furthermore, maternal risk stratification models that take into account age, comorbidities, and fat distribution have been put forward to aid in personalized care [18]. Nevertheless, the adoption of such models in everyday clinical settings is still low, and more high-quality evidence is required to confirm their effectiveness and scalability [19].

Due to the broad impact of maternal obesity on both short and long-term health of the mother and the offspring, a systematic review of the existing evidence concerning the management of maternal obesity during pregnancy is crucial [20]. Although several studies and reviews addressed individual aspects of this problem, there is still a need for an integrated synthesis to compile relevant findings regarding antenatal care strategies, maternal and neonatal outcomes, as well as long-term health effects. This systematic review seeks to address that gap by appraising and synthesizing evidence from top-quality peer-reviewed published studies between 2015 and 2024. By doing so, it aims to guide clinical application, discover deficits of current knowledge, and contribute to the development of standardized and multidisciplinary care.

This review is conducted in accordance with the Preferred Reporting Items for Systematic Reviews and Meta-Analyses (PRISMA) guidelines for methodological robustness and openness. It involved an extensive search of databases like PubMed, Embase, Scopus, and Web of Science and included only those studies satisfying strict inclusion criteria, such as a focus on antenatal management, maternal outcomes, and long-term health effects. By analyzing both clinical interventions and outcome data, the review presents a solid foundation for assessing the current state of maternal obesity management and opens new avenues for research and policy directions for the future.

## Review

Methodology

**Figure 1:**
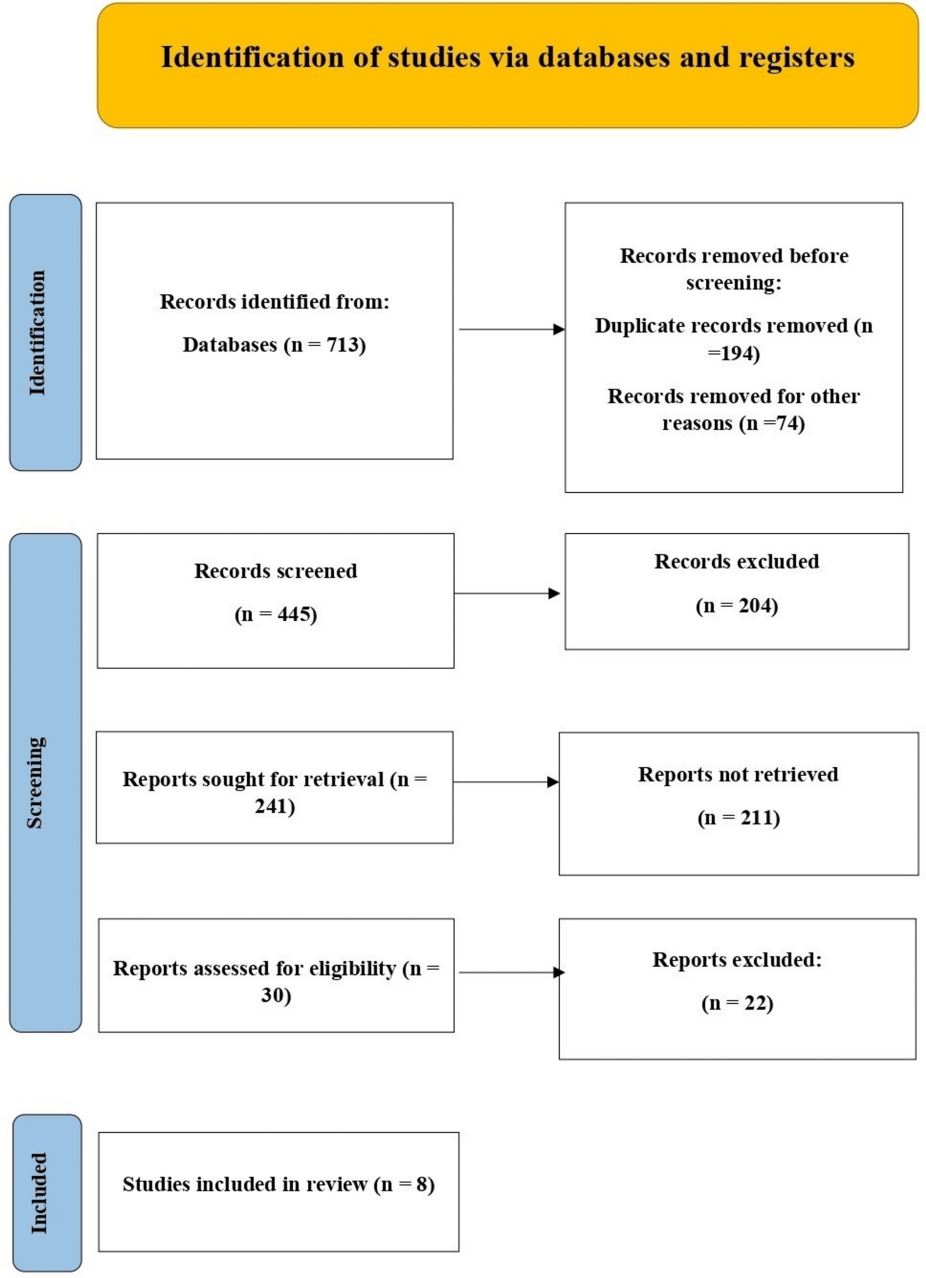
PRISMA flowchart PRISMA: Preferred Reporting Items for Systematic Reviews and Meta-Analyses

This systematic review aimed to review the management of maternal obesity in pregnancy, which included antenatal care intervention, outcome of antenatal care, and long-term effects on maternal health. The review was conducted in line with the PRISMA guidelines to maintain transparency and methodological soundness. To identify relevant studies, a comprehensive search was conducted using Boolean operators combining key concepts related to maternal obesity and pregnancy outcomes. The search strategy included the following terms: (“maternal obesity” OR “obesity in pregnancy”) AND (“antenatal care” OR “antenatal management” OR “prenatal care”) AND (“pregnancy outcomes” OR “perinatal outcomes” OR “maternal outcomes” OR “neonatal outcomes” OR “birth outcomes”) AND (“long-term effects” OR “long-term implications” OR “maternal health” OR “offspring health”) AND (“gestational diabetes” OR “hypertensive disorders” OR “preterm birth” OR “cesarean delivery” OR “large for gestational age” OR “congenital anomalies”) AND (“guidelines” OR “screening” OR “weight gain” OR “lifestyle counseling” OR “interventions” OR “predictive model”). Continuity and consistency were maintained through the entire research process. The conduct of the literature search, the criteria for identifying studies for selection, the extraction of data, and the steps toward quality assessment were well-structured and meticulously followed. The detailed process of identification, screening, and inclusion of studies is illustrated in Figure 1.

Search Strategy and Data Sources

A thorough search of the literature was conducted through electronic databases such as PubMed, Embase, Scopus, and Web of Science. For literature identification and relevant articles, Google Scholar was used. The scope was restricted to peer-reviewed studies published between January 2015 and April 2024. Keywords used in multiple combinations were as follows: “maternal obesity”, “pregnancy outcomes”, “antenatal care”, “gestational weight gain”, “maternal health”, “obstetric complications”, “long-term implications”. Boolean operators and filters specific to the databases were used to limit the results to studies conducted among human participants and articles published in English.

Inclusion and Exclusion Criteria

The inclusion criteria were as follows: (1) studies involving women with a pre-pregnancy BMI ≥30 kg/m²; (2) those focusing on the management of obesity in pregnancy, including clinical guidelines, interventions, or antenatal care, and (3) those assessing maternal or neonatal outcomes or long-term maternal health consequences. (4) Studies were excluded if they involved non-pregnant populations, animal models, or if they omitted data on relevant outcomes.

Study Selection Process

A total of 713 records were identified via database searches. After removing 194 duplicates and 74 records for other reasons, 445 records were screened. Of these, 204 were excluded based on titles and abstracts. Full texts were sought for 241 records, but 211 could not be retrieved. Thirty full-text articles were assessed for eligibility, of which 22 were excluded for not meeting the inclusion criteria. Ultimately, eight studies were included in the final analysis.

Data Extraction

A standardized data extraction form was created and employed to capture key characteristics of the studies such as the authors, publication year, country of study, study design, sample size, characteristics of the population, maternal BMI grouping, interventions, strategies for antenatal care, maternal and neonatal outcomes, as well as long-term maternal health implications of obesity. Effect sizes such as odds ratios (OR), relative risks (RR), and confidence intervals (CI) were also extracted if available. The extraction was performed for the independent research by two reviewers, and discrepancies in data were resolved via discussion.

Quality Assessment

The quality of the included studies was determined through the Newcastle-Ottawa Scale (NOS) for observational studies and the Cochrane Risk of Bias Tool for randomized trials. Examples of criteria included the choice of the study groups, comparability of cohorts, and outcome measurement. Studies with a score of 6 and above on the NOS were regarded as moderate to high quality studies. All the studies incorporated herein achieved this threshold, reinforcing the reliability of their findings.

Data Synthesis and Analysis

Given the study design, population sample, interventions, and outcomes heterogeneity, a narrative synthesis approach was used. Some of the key themes that emerged from the analysis were the complexity of managing maternal obesity, diversity in clinical guidelines, and the broad range of maternal and fetal risks from obesity in pregnancy. Of note was the close observation of recurrent complications, including GDM, preeclampsia, cesarean delivery, preterm birth, and neonatal ICU admissions. Future cardiometabolic diseases in mothers were also brought to the fore.

Ethical Considerations

This study utilized secondary analysis of published data, and hence no ethical approval was required. Nevertheless, all the selected studies were peer-reviewed and met their respective ethical standards relating to informed consent and institutional review board (IRB) approvals.

Limitations

Some limitations exist, although attempts were made to ensure a comprehensive approach. Non-English language studies were excluded, and thus, international findings may have been omitted. Furthermore, differences in clinical practices between countries might have an impact on generalizability. Finally, the review incorporates studies performed until 2024, and with the rapidity of the changes in clinical guidelines, the standard of care may dictate the same even after this period.

Results

Overall, eight peer-reviewed studies published from 2015 to 2024 were included for this systematic review. The studies selected involved three systematic reviews, three cohort studies, and two narrative reviews. The studies emanated from diverse geographical regions and examined different maternal and neonatal outcomes as a result of maternal obesity. Repeated patterns were evident in the literature included. Maternal obesity was commonly reported as being linked with a higher incidence of GDM, hypertensive disorders during pregnancy, preterm birth, and cesarean section. Some of these correlations were quantified in various studies, and one estimated the percentage of adverse outcomes related to obesity. Neonatal outcomes, including respiratory distress syndrome, sepsis, congenital anomalies, and large for gestational age (LGA) births, were observed with increased frequency among the children of mothers with obesity.

Longitudinal studies showed cross-sections of maternal obesity and lifelong cardiometabolic risk in mothers, along with neurodevelopmental and metabolic problems in offspring. Studies identified the existence of heterogeneity in the risk profiles of obese pregnant women, where factors include maternal age, ethnicity, and pre-existing comorbidities like, among others, type 1 diabetes and central adiposity. Variation in clinical practice guidelines and implementation of antenatal care strategies was also recorded. Several studies found gaps in the existing management protocols in terms of their consistency and clarity. As a response, certain literature outlined the antenatal interventions, including the targeted screening, lifestyle counseling, and multidisciplinary monitoring as commonly suggested strategies, though the quality and usage of these strategies differed from setting to setting. A concise summary of the study characteristics, key findings, and addressed outcomes is presented in Table 1 below.

**Table 1:** Summary of included studies GDM: gestational diabetes mellitus; GWG: gestational weight gain; LGA: large for gestational age; PTB: preterm birth

Study	Year	Study type	Key findings	Outcomes addressed
Lim and Mahmood (2015) [21]	2015	Narrative review	Maternal obesity increases the risk of obstetric complications and perinatal morbidity	Maternal and fetal complications
Mariona (2016) [22]	2016	Narrative review	Highlights inconsistent guidelines and a broad spectrum of maternal and fetal risks	GDM, hypertension, bariatric care, long-term risks
Carlson et al. (2018) [23]	2018	Systematic review	Reviewed clinical strategies for antepartum care in obese women	GDM, sleep apnea, mental health, imaging, delivery
Yang et al. (2019) [24]	2019	Cohort study	Quantified risk of adverse outcomes; 36% of preeclampsia cases attributable to obesity	GDM, preeclampsia, cesarean, PTB, LGA
Grieger et al. (2021) [25]	2021	Narrative review	Links obesity and GWG with cardiometabolic outcomes in mothers	Postpartum cardiometabolic disease, GDM, preeclampsia
Fakhraei et al. (2022) [26]	2022	Systematic review	Identified predictors of complications in obese pregnant women	GDM, preterm birth, low birthweight, stillbirth
Dinsmoor et al. (2023) [27]	2023	Cohort study	Morbid obesity is associated with a 33% higher neonatal morbidity	Composite neonatal morbidity
Alves et al. (2024) [28]	2024	Narrative review	Emphasizes the intergenerational impact of obesity during pregnancy	Cardiometabolic and neurodevelopmental offspring outcomes

Together, the studies show the need for the urgency of implementing standardized guidelines for the management of maternal obesity antenatally. Although all studies discussed increased perinatal risks, some also highlighted the long-term health consequences, affecting the mother and baby post-delivery. One of the recurring themes was the need for early detection and interventions that are specialized, supported by multi-disciplinary care frameworks. Furthermore, risk stratification strategies are being increasingly seen as necessary for personalizing care and maximizing outcomes.

Discussion

This systematic review synthesizes the existing evidence regarding the management of maternal obesity in pregnancy, antenatal care strategies, outcomes associated with this, and the long-term effects on maternal health. The eight high-quality studies included have a range of methodologies (including systematic and narrative reviews, as well as cohort studies), so that a holistic picture of the multifactorial complexity and considerations in the management of obesity during pregnancy is provided. Taken together, they reinforce the public health relevance of maternal obesity, with important consequences on pregnancy outcome and maternal and offspring health in the future.

One of the common themes across the studies is the consistency in associating maternal obesity with deleterious pregnancy outcomes. Several research works have noted a greatly elevated risk of GDM, hypertensive disorders including preeclampsia, cesarean section, as well as preterm birth in obese women [29]. These findings have clinical implications since they suggest that different antenatal care and early identification of risks are needed. It is observed from the data that obesity not only leads to the incidence of these conditions but also can aggravate their severity, thereby increasing rates of maternal and neonatal morbidity.

Another area of concern emerging from the studies included is that of neonatal outcomes. Increased odds of neonatal complications such as respiratory distress syndrome, neonatal intensive care unit (NICU) admission, and sepsis are associated with maternal morbid obesity [30]. Similarly, the studies reveal that maternal obesity is linked with congenital anomalies and LGA infants. These findings are in line with the previous literature showing that the intrauterine environment of obese pregnancies may predispose neonates to immediate and lifelong health hazards.

In addition to the immediate pregnancy and neonatal outcomes, the long-term health consequences for the mothers and offspring are a focal matter of concern. Certain studies identify maternal obesity during pregnancy as a precursor for later cardiometabolic abnormalities in mothers, such as type 2 diabetes, hypertension, and cardiovascular disease. In addition, there was the intergenerational transfer of risk, with children born to obese mothers having a higher likelihood of becoming obese as well as developing metabolic syndrome and neurodevelopmental issues in adulthood. These findings highlight the need for accelerated interventions aimed at maternal obesity to extend beyond short-term outcomes during the perinatal period and break the chains of obesity and chronic diseases for future generations.

One of the insights gained from this review is the variation, and in many instances, the inadequacy of the existing antenatal care guidelines for treating maternal obesity. Few studies directly refer to discrepancies in clinical recommendations in terms of adequate gestational weight gain, screening intervals, nutritional counseling, and physical activity. This lack of standardized care pathways indicates the absence of evidence-based guidelines and the difficulty in translating available knowledge to clinical practice. Besides, a lack of sufficient provider training and the stigma related to obesity could partly undermine effective management. This requires a more coordinated approach that involves revised guidelines, education of providers, and incorporation of obesity care into the service of routine prenatal care.

Adequate antenatal treatment was seen as critical in controlling undesirable outcomes. The significance of multidisciplinary methods involving dieticians, endocrinologists, professionals in mental health, as well as obstetricians, being on the same page has been discussed. Lifestyle interventions, particularly those initiated at early stages of pregnancy or preconception, demonstrate potential in achieving better outcomes; however, more rigorous studies are required to consolidate these findings. Risk stratification tools provide a pragmatic approach to individualizing care based on age, ethnicity, and other comorbidities such as type 1 diabetes or central adiposity. Such focused treatment may contribute to boosting clinical effectiveness and resource redistribution.

Despite this review's numerous strengths, such as its robust approach and the inclusion of high-quality studies, there are certain limitations that should be acknowledged. The exclusion of non-English publications may have neglected international insights, especially from low- and middle-income countries (LMICs) where obesity among mothers is also increasing. Moreover, the included research had varying designs and a regional context that can influence generalizations. The dynamic nature of clinical practice guidelines also implies that some recommendations in them will be outdated when new evidence emerges.

Finally, this review highlights maternal obesity as a leading risk factor for perinatal adverse outcomes and long-term maternal and offspring health complications. The evidence demonstrates the requirement for uniform, evidence-based antenatal care protocols, early risk identification, and use of multidisciplinary interventions to optimize outcomes. Further studies should be conducted to design and implement integrated models of care and risk prediction tools for use during personalized antenatal care. The need to prevent maternal obesity effectively begins with addressing maternal and neonatal outcomes, but is carried forward into a public health issue with long-ranging intergenerational impact.

## Conclusions

The findings of this systematic review confirm that maternal obesity has a serious impact on a wide range of perinatal and long-term health problems of mother and child. Key complications include gestational diabetes, hypertensive disorders, preterm birth, and cesarean delivery, where adverse implications go beyond the postpartum stage to future maternal health, including cardiovascular disease and type 2 diabetes, among others. This is significant for obstetric care, especially concerning metabolic and neurodevelopmental disorders in the offspring, emphasizing the long-term consequences of maternal obesity. Despite growing awareness, the antenatal guidelines are still not uniform, clinical practice is frequently affected, and resources are scarce. These issues can be mitigated with the early identification of high-risk women using risk stratification models and with individualized, multidisciplinary care approaches. Lifestyle interventions (including dietary counseling, physical exercise, and psychological intervention) are promising but underexploited avenues. The standardization of care protocols and the incorporation of obesity management into routine prenatal care should become a priority. Future research should be performed on scalable care models and evidence-based interventions that can be used in diverse healthcare settings. Maternal obesity management is not just important for short-term pregnancies but also for the overall health of future generations.
